# One Stone Four Birds: A Novel Liposomal Delivery System Multi-functionalized with Ginsenoside Rh2 for Tumor Targeting Therapy

**DOI:** 10.1007/s40820-020-00472-8

**Published:** 2020-06-16

**Authors:** Chao Hong, Jianming Liang, Jiaxuan Xia, Ying Zhu, Yizhen Guo, Anni Wang, Chunyi Lu, Hongwei Ren, Chen Chen, Shiyi Li, Dan Wang, Huaxing Zhan, Jianxin Wang

**Affiliations:** 1 Department of Pharmaceutics, School of Pharmacy, Fudan University & Key Laboratory of Smart Drug Delivery, Ministry of Education, Shanghai, 201203 People’s Republic of China; 2 Institute of Tropical Medicine, Guangzhou University of Chinese Medicine, Guangzhou, 510006 People’s Republic of China; 3 Institute of Clinical Pharmacology, Guangzhou University of Chinese Medicine, Guangzhou, 510006 People’s Republic of China; 4 School of Pharmacy, Shanghai Jiao Tong University, Shanghai, 200240 People’s Republic of China; 5 Shanghai Ginposome Pharmatech Co., Ltd, Shanghai, 201600 People’s Republic of China; 6 Institute of Integrated Chinese and Western Medicine, Fudan University, Shanghai, 200040 People’s Republic of China

**Keywords:** Ginsenoside Rh2, Liposomes, Cholesterol, Multifunction, Tumor targeting

## Abstract

Liposomes hold great potential in anti-cancer drug delivery and the targeting treatment of tumors. However, the clinical therapeutic efficacy of liposomes is still limited by the complexity of tumor microenvironment (TME) and the insufficient accumulation in tumor sites. Meanwhile, the application of cholesterol and polyethylene glycol (PEG), which are usually used to prolong the blood circulation and stabilize the structure of liposomes respectively, has been questioned due to various disadvantages. Herein, we developed a ginsenoside Rh2-based multifunctional liposome system (Rh2-lipo) to effectively address these challenges once for all. Different with the conventional ‘wooden’ liposomes, Rh2-lipo is a much more brilliant carrier with multiple functions. In Rh2-lipo, both cholesterol and PEG were substituted by Rh2, which works as membrane stabilizer, long-circulating stealther, active targeting ligand, and chemotherapy adjuvant at the same time. Firstly, Rh2 could keep the stability of liposomes and avoid the shortcomings caused by cholesterol. Secondly, Rh2-lipo showed a specifically prolonged circulation behavior in the blood. Thirdly, the accumulation of the liposomes in the tumor was significantly enhanced by the interaction of glucose transporter of tumor cells with Rh2. Fourth, Rh2-lipo could remodel the structure and reverse the immunosuppressive environment in TME. When tested in a 4T1 breast carcinoma xenograft model, the paclitaxel-loaded Rh2-lipo realized high efficient tumor growth suppression. Therefore, Rh2-lipo not only innovatively challenges the position of cholesterol as a liposome component, but also provides another innovative potential system with multiple functions for anti-cancer drug delivery.

**Electronic supplementary material:**

The online version of this article (10.1007/s40820-020-00472-8) contains supplementary material, which is available to authorized users.

## Highlights

- A ginsenoside Rh2-based multifunctional liposome system (Rh2-lipo) was innovatively developed.
- Rh2-lipo not only innovatively challenges the position of cholesterol as a liposome component, but also provides another innovative potential system with multiple functions for anti-cancer drug delivery.

**Electronic supplementary material:**

The online version of this article (10.1007/s40820-020-00472-8) contains supplementary material, which is available to authorized users.

## Graphical abstract

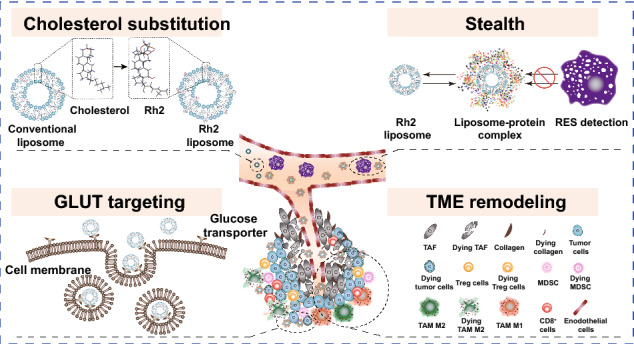

## Introduction

Nanoparticle-based drug delivery is one of the well-established nanotechnologies which has the potential to address many limitations encountered in current cancer therapies [1]. Liposome, a specific type of nano-carrier that has been successfully applied in drug delivery system, has passed into clinical use with different formulations [2, 3]. With its enhanced permeability and retention effect, liposomes can deliver the therapeutic agent preferentially to the tumor site with reduced systemic toxicity compared to free drug and thus improve the therapeutic efficacy of anti-cancer agents [4].

However, despite various advantages mentioned above, the clinical application of liposomes has been limited due to the following reasons: (1) Tumor cells can recruit many type of endogenous cells, such as immune inflammatory cells, to develop an immunodeficiency environment for self-protection and maintain the multistage growth and development of tumor [5]. Since the complexity of tumor environment, liposomes that carry single chemotherapy agent will not provide satisfying therapeutic efficacy. A delivery system that simultaneously targets tumor cells and tumor microenvironment (TME) is in desperate need. (2) Although polyethylene glycolation (PEGylation) can prolong the circulation time of liposomes in the blood, it reduces uptake by target cells and can be highly immunogenic, which would dim its application as a potential delivery system for anti-cancer agents in future [6, 7]. (3) The application of ligand-targeted liposomes often results in lower targeting efficiency and higher systemic toxicity than expected. Therefore, none of the active targeting liposomal drug delivery systems has been fully approved by FDA at present [8]. (4) Cholesterol, one of the indispensable components of liposomes, has been questioned in clinics due to religion tradition and vegetarianism related issues. It has also been found that cholesterol is able to regulate serum lipoprotein level to induce ‘complement-mediated pseudoallergic reaction,’ leading to pulmonary hypertension and other cardiopulmonary side effects [9, 10]. For example, liposomal formulations of doxorubicin (Doxil^®^) and amphotericin B (Ambisome^®^) are increasingly used in the treatment of cancer and other diseases due to efficient in improving the therapeutic efficacy. However, these liposomes can cause an immediate hypersensitivity reaction in a relatively large number (up to 7%) of patients [11–16]. According to the literature, the symptoms include cardiopulmonary distress such as dyspnea, tachypnea, hypertension/hypotension, chest pain, and back pain, which could be partly attributed to the enrichment of cholesterol in liposomes [9, 10]. Though phytosterol has been confirmed to exert similar effect on phospholipid bilayer and used as an alternative to prepare non-cholesterol liposomes, the anti-cancer efficacy of such delivery system has not been fully investigated [17, 18]. Considering the challenges mentioned above, it is of great importance to design a novel liposomal delivery system that could effectively address these challenges once for all.

**Fig. 1:**
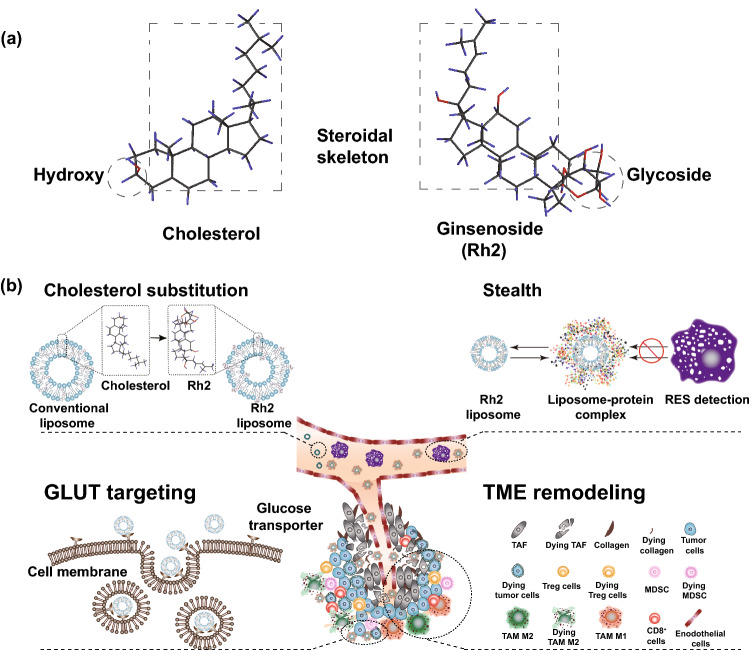
Rational design of a multifunctional Rh2-lipo for tumor targeting therapy. **a** Chemical structure of cholesterol and ginsenoside. **b** In Rh2-lipo, cholesterol was substituted by Rh2, which works as membrane stabilizer, long-circulating stealther, targeting ligand of GLUT and chemotherapy adjuvant at the same time, empowering liposome with multiple functions including long circulation, active targeting, TME remodeling, and tumor cell killing capacity

Ginsenosides are effective components extracted from ginseng. They are composed of hydrophilic glycoside chains and hydrophobic aglycones, in which the aglycones share the similar steroid structure with cholesterol (Fig. 1a, Rh2, as a representative of ginsenoside) [19]. With electron spin resonance, micro-thermal technology, Raman spectroscopy, and differential scanning calorimetry, researchers have studied the interaction of different ginsenosides (Rh2, Rb1, Re, Rf, and Rg1) with various phospholipids and demonstrated that ginsenosides have the similar ability as cholesterol to improve physical and chemical properties of phospholipid bilayer [20, 21]. Evidence has also indicated that ginsenosides could possibly be a reliable alternative for cholesterol as a component of liposomes. Yin et al. [22] investigated the interactions between 1,2-distearoyl-sn-glycero-3-phosphocholine (DSPC) and AD1, a ginsenoside extracted from *Panax notoginseng*. Compared with pure DSPC, AD1, and DSPC mixture had favorable geometrical interactions and increased stability at the interface, highlighting that ginsenoside/phospholipid interactions could be advantageous for drug cargo system. Lipid packing order of phospholipid bilayer, size, and surface status of liposomes could be significantly changed with the insertion of ginsenoside, which would impact the in vivo fate of liposomes to some extent. Moreover, as confirmed substrates of glucose transporter 1 (GLUT1) and sodium-coupled glucose co-transporter 1 (SGLT1) [23, 24], ginsenosides would also facilitate the targeting of liposomes to tumors overexpressing those transporters [25], and exert improved therapeutic effect.

Ginsenosides have prominent anti-tumor activities and are commonly used in combination with first-line anti-cancer drugs to enhance efficiency and reduce adverse reactions of chemotherapeutics [26]. 20(S)-Rh2, one of the ginsenosides that have been fully investigated [27], triggers multiple signal transduction pathways involving TGF-b1/Smad, STAT3, NF-κB, etc. [28, 29], and exerts remarkable effects including anti-angiogenesis, anti-inflammation, anti-proliferation, anti-oxidation, and immunological response enhancement. With such diverse anti-tumor activities, Rh2 has been proved having synergistic anti-tumor effects with conventional chemotherapeutic agents [30, 31] and has great potential to be a TME modifier to enhance efficacy of chemotherapies. However, since Rh2 degrades in gastrointestinal tract and the blood and cannot reach tumor site with anti-cancer drug simultaneously, its effect is greatly restricted.

Based on the outstanding physicochemical properties and anti-cancer activities of ginsenoside Rh2, in order to address the limitations faced by conventional liposomes, we creatively developed a novel nano-carrier, denoted as ginsenoside Rh2 liposome (Rh2-lipo). As illustrated in Fig. 1b, cholesterol was substituted by Rh2 in Rh2-lipo, where Rh2 worked as both membrane stabilizer and chemotherapy adjuvant. Different with the ‘wooden’ conventional liposomes, Rh2-lipo is a much more brilliant carrier with multiple functions including long circulation, active targeting, TME remodeling, and tumor cell killing capacity.

## Experimental

### Materials

Paclitaxel (PTX) was obtained from Dalian Meilun Biotechnology Co., Ltd. (Dalian, China). Egg yolk lecithin (EYPC) and cholesterol were purchased by A.V.T pharmaceutical Co., Ltd. (Shanghai, China). Lipusu^®^ (Lipusu) and ginsenoside Rh2 were provided by Shanghai Ginposome Pharmatech Co., Ltd. (Shanghai, China). 5-Carboxyfluorescein (FAM), hoechst 33342, and near-infrared dye 1,1′-Dioctadecyl-3,3,3′,3′-tetramethylindodicarbocyanine perchlorate (DID) were received from Fanbo Biochemicals (Beijing, China). The 5-doxyl-stearic acid (5-DSA), 16-doxyl-stearic acid (16-DSA), and tetrazolium-based colorimetric (MTT) were purchased from Sigma (St. Louis, MO, USA). mPEG_2000_-DSPE was obtained by NOF Co. (Tokyo, Japan).

4T1 cells were obtained from the Cell Bank of the Chinese Academy of Sciences (Shanghai, China). The cells were cultured in RPMI 1640 medium supplemented with 10% fetal bovine serum (Gemini), 2 mmol L^−1^ glutamine, and 1% penicillin/streptomycin at 37 °C in a humidified 5% CO_2_ incubator (Thermo Scientific, USA).

BALB/c and ICR mice (18–20 g) were purchased from Shanghai SLAC Laboratory Animal Co., Ltd. (Shanghai, China). All animal experiments were performed in accordance with the principles of care and use of laboratory animals. The protocol of animal experiments was approved by the Animal Experimentation Ethics Committee of Fudan University.

### Preparation and Characterization of Liposomes

Liposomes were prepared by a thin-film hydration method. Cholesterol-liposome (C-lipo) was made of EYPC and cholesterol (10:3, mass ratio), while Rh2-lipo was prepared with a same lipid composition of EYPC/Rh2 = 10:3. Briefly, 6 mg cholesterol or Rh2 and 20 mg EYPC were dissolved in 1 mL CH_3_CH_2_OH and CHCl_3_ (1:1), followed by rotary evaporation to form a lipid film using a ZX-98 rotary evaporator (LOOYE, China) at 50 °C. After the thin film was hydrated with 1 mL 5% glucose solution at 50 °C for 30 min, the liposomal suspension was then subjected to a probing sonication process in an ice bath for 5 min at 300 W with a sequence of 5 s of sonication and 5 s of rest using a sonicator (Sonics & Materials, Inc., 20 kHz).

Particle size and ζ-potential of all liposomes were measured with a dynamic light scattering (DLS) detector (Zetasizer, Nano-ZS, Malvern, UK). The morphology of PTX loaded liposomes was detected by transmission electron microscope (TEM) (Tecnai G2 F20 S-Twin, FEI, USA). The drug encapsulation efficiency (EE) and loading efficiency (LE) of PTX were measured using a previously reported method [32]. In brief, the concentration of PTX retained in liposomes was determined after removing the precipitated PTX by filtration through a 0.8 μm membrane filter. Then, EE and LE of PTX were calculated using the following equations:

$$ {\text{EE}}\,\left( \% \right) = \frac{{{\text{Amount}}\;{\text{of}}\;{\text{PTX}}\;{\text{loaded}}\;{\text{in}}\;{\text{liposomes}}}}{{{\text{Total}}\;{\text{amount}}\;{\text{of}}\;{\text{PTX }}}} \times 100\% $$

$$ {\text{LE}}\,\left( \% \right) = \frac{{{\text{Amount}}\;{\text{of}}\;{\text{PTX}}\;{\text{loaded}}\;{\text{in}}\;{\text{liposomes}}}}{{{\text{Total}}\;{\text{amount}}\;{\text{of}}\;{\text{materies }}}} \times 100\% $$

### Electron Paramagnetic Resonance Analysis

Electron paramagnetic resonance (EPR) techniques were used to monitor the molecular dynamics of lipid bilayer modulated by Rh2 via observing the changes in the spin tropic movement of an unpaired electron. Spin markers 5-DSA and 16-DSA, who containing a radical group at position 5 or 16 of the alkyl chain, respectively, were used to reflect packing order in the polar head region or hydrophobic region of the lipid bilayer. A series amount of cholesterol or Rh2 was encapsulated in 5-DSA, and 16-DSA-labeled liposomes were prepared by the same method described above with a mass ratio of EYPC/cholesterol or Rh2/DSA = 10: (0, 4, 8, 12, 16): 0.01. The experiments were performed on a Bruker EMX spectrometer (Bruker, Rheinstetten, Germany). The test parameters were set as follows: microwave power 15 mW, sweep time 10 s, center field 3500 G, and sweep width 100 G. The value of isotropic hyperfine coupling constant *a*_*0*_ and molecular order parameter S can be calculated from the recorded spectral as follows:

$$ a_{0} = \left( {A_{\rm{max}} + 2A_{\rm{min}} } \right)/3 $$

$$ S = 0.5407 \times \left( {A_{\rm{max}} - A_{\rm{min}} } \right)a_{0} , $$

where line widths 2*A*_max_ and 2*A*_min_ represented the splittings between the outer peaks and the inner peaks in the simulated spectra [33].

### Stability of Blank Rh2-lipo and PTX-Rh2-lipo

The stability of C-lipo, Rh2-lipo, PTX-C-lipo, and PTX-Rh2-lipo in phosphate buffer saline (PBS) was conducted at 4 °C. Briefly, 1 mL liposomes were dispersed in 9 mL 1 × PBS, and the average size and polydispersity index (PDI) were measured by DLS every day for 7 days. The PTX content remaining as loaded in liposomes was also tested every day to determine the change of EE of PTX liposomes during the storage period [32]. Each sample has three duplicates.

### Pharmacokinetics Study

The in vivo circulation time of the Rh2-lipo was determined as previously reported [34]. DID dye was applied to label C-lipo, PEG-C-lipo, and Rh2-lipo, which was then injected into ICR mice through the tail vein (10 mg kg^−1^), and each sample has three duplicates. Then, 50 μL of blood samples was collected from the submaxillary vein at 2 min, 5 min, 15 min, 30 min, 1 h, 3 h, 6 h, 12 h, and 24 h. The collected blood samples were diluted with 50 μL of 1 × PBS in a 96-well plate before fluorescence measurement (644/665 nm). All PK parameters were calculated using PKSolver following the instructions.

### Protein Corona (PC) Preparation

Liposome–protein complexes were prepared by incubating liposomes with plasma (1:1 v/v) at 37 °C for 1 h. Experimental conditions (i.e., plasma concentration, temperature and incubation time) were chosen according to previous investigations [35–37]. After incubating, liposome–protein complexes were isolated by centrifugation for 15 min at 14,000 rpm. Then, the pellets were washed three times with PBS to remove unbound proteins.

### Sodium Dodecyl Sulfate Polyacrylamide Gel Electrophoresis (SDS-PAGE) Experiments

The composition of the PC was investigated by SDS-PAGE firstly. Five microliters of liposome–protein complexes was resuspended in 20 μL of loading buffer 1 × (10 μL βME per 1 ml loading buffer) and boiled for 5 min at 100 °C. Identical volumes (10 μL) of each sample were loaded and separated by 10% SDS-PAGE (running at 100 V for about 100 min). Then, the gels were obtained and dyed by coomassie brilliant blue (CBB) R250 overnight. Once all the protein bands started to clearly appear, the staining solution was removed, and the stop solution (acetic acid 10%, methanol 45%) was added. Gel images were processed by means of customized software. Semi-quantitative densitometry analysis of the protein bands was analyzed on ImageJ software.

### Nano-Liquid Chromatography–Mass Spectrometry (Nano-LC–MS/MS) Analysis

To further identify the composition of PC, a nano-LC–MS/MS analysis was carried out. Firstly, each sample was allowed to proceed at 56 °C for 1 h in an appropriate volume of 10 mM dithiotreitol in 25 mM NH_4_HCO_3_ and the alkylation was allowed to proceed in the dark for 45 min at room temperature. After been mixed with an appropriate volume of 12.5 ng μL^−1^ trypsin, the digestion was carried out at 37 °C overnight. An appropriate volume of 60% acetonitrile, 0.2% trifluoroacetic acid, was added. The sample was desalted with a C_18_ZipTip (Millipore), and half of the eluate was analyzed with nano-LC–MS/MS.

The samples resuspended with 30 μL solvent C (C: water with 0.1% formic acid) were separated by nano-LC and analyzed by online electrospray tandem mass spectrometry. The experiments were performed on a Nano-ACQUITY UPLC system (Waters Corporation, Milford, MA) connected to a Q-Exactive mass spectrometer (Thermo Fisher Scientific, MA, USA) equipped with an online nano-electrospray ion source. Ten microliters of peptide sample was loaded onto the trap column (Thermo Scientific Acclaim PepMap C18, 100 μm × 2 cm), with a flow of 10 μL min^−1^ for 3 min and subsequently separated on the analytical column (Acclaim PepMap C18, 75 μm × 15 cm) with a 90 min linear gradient, from 5% D (D: acetonitrile with 0.1% formic acid) to 55% D. The column was re-equilibrated at initial conditions for 10 min. The column flow rate was maintained at 300 nL min^−1^. The electrospray voltage of 2 kV versus the inlet of the mass spectrometer was used. The mass spectrometer was run under data-dependent acquisition mode, and automatically switched under MS and MS/MS mode. MS1 mass resolution was set as 70 K with m/z 300-1800, and MS/MS resolution was set as 17.5 K under HCD mode. The dynamic exclusion time was set as 10 s.

### In Vivo Animal Imaging

The 4T1-orthotopic-bearing tumor mouse model was established to study the targeting effect of Rh2-lipo. Briefly, 10^5^ 4T1 breast cancer cells suspended in PBS were injected into the left inguinal gland (100 µL injection volume) to initiate orthotopic 4T1 breast tumors in BALB/c mice. When the volume of tumors reached approximately 100 mm^3^, tumor-bearing mice were intravenously injected via the tail with DID-loaded Rh2-lipo (20 mg kg^−1^). The same amount of DID-loaded C-lipo solution was injected as control. The distribution of fluorescence was observed at predetermined time points (2, 4, 8, 12, and 24 h) using an in vivo imaging system (IVIS Spectrum, Caliper, USA). Twenty-four hours later, the mice were sacrificed after heart perfusion with saline. Then, the tumors and peripheric organs were harvested and imaged.

### In Vitro Cellular Uptake

An in vitro cellular uptake experiment was performed to further investigate the targeting mechanism of Rh2-lipo. 4T1 cells were seeded at a density of 2 × 10^5^ cells/well in 24-well plates. After being incubated overnight, FAM-loaded C-lipo and Rh2-lipo were added to 4T1 cells with FAM final concentration of 500 ng mL^−1^ at 37 °C for 4 h. Then, 4T1 cells were trypsinized and washed for three times with cold PBS (pH 7.4), subsequently analyzed using a flow cytometer (FACS Calibur, BD Biosciences, USA). To investigate the mechanism of liposome uptake, we preincubated cells with 20 mM glucose for 60 min. After incubation, the solution was removed and the cells were washed three times with cold PBS (pH 7.4). Then, the follow-up work was the same as above. For the qualitative study, after incubation, 4T1 cells were stained with DAPI, washed three times, and visualized using a confocal microscope (Carl Zeiss, Germany).

### Cytotoxicity Assay

The MTT assay could determine the living cells in vitro selectively; thus, it was used to assess the cytotoxicity of the PTX-loaded Rh2-lipo here. Briefly, 4T1 cells were incubated with free PTX, PTX-C-lipo, PTX-Rh2-lipo, Rh2, and Rh2-lipo (Rh2 concentrations used in free Rh2 and Rh2-lipo groups were consistent with the corresponding Rh2 concentration in PTX-Rh2-lipo) for 48 h, followed by a MTT assay according to the manufacturer’s protocol. The cell viability was calculated as the equation:

$$ {\text{Cell}}\;{\text{viability}}\,\left( \%  \right) = \frac{{{\text{OD}}_{{{\text{experimental}}\;{\text{group}}}} }}{{{\text{OD}}_{{{\text{control}}\;{\text{group}}}} }} \times 100\%  $$

The IC_50_ values were calculated using GraphPad Prism software.

### Apoptosis Assay

The 4T1 cells in logarithmic growth phase were digested with trypsin, seeded at appropriate density, and incubated for 12 h. After the cells were treated with free PTX, PTX-C-lipo, PTX-Rh2-lipo, Rh2, and Rh2-lipo (PTX concentration was 1 μg mL^−1^; Rh2 concentration used in free Rh2 and Rh2-lipo groups was consistent with the corresponding Rh2 concentration in the PTX-Rh2-lipo) for 48 h, the apoptosis cells were measured with flow cytometry using Annexin V-FITC/PI double staining assay. The cells were classified as normal (Annexin V-, PI-), early apoptotic (Annexin V + , PI-), and late apoptotic (Annexin V + , PI +).

### In Vivo Anti-tumor Effect and Structure Remodeling in TME

The anti-tumor efficacy of the PTX-Rh2-lipo was evaluated by monitoring tumor growth in 4T1-orthotopic-bearing tumor BALB/c mice model as described above. When the volume of tumors reached approximately 100 mm^3^, the mice were randomly assigned to each group. The mice were administered 200 µL Lipusu and PTX-Rh2-lipo with equivalent PTX content at 10 mg/kg body weight, while Rh2 and Rh2-lipo with Rh2 content of 30 mg/kg body weight (200 µL). The same volume of PBS was injected as control. The formulations were intravenously injected via tail veins every other day for 21 days. The tumor size and body weight were measured every three days and calculated as length × width^2^/2. The mice were then sacrificed, and the tumors as well as other principal organs were excised carefully for histological examination.

To further evaluate the structure change in TME after treatment of Rh2-lipo formulation, the tumors were fixed with 4% (v/v) paraformaldehyde in PBS (pH 7.4) and sectioned into 5 μm slices. Apoptotic and non-apoptotic cells in tumor tissues were histologically evaluated with the terminal deoxynucleotidyltransferase-mediated nick end labeling (TUNEL) assay, using a commercial apoptosis detection kit (Promega, Madison, WI). Tumor-associated fibroblast (TAF) and vessels were characterized by rabbit anti-mouse α-SMA and rabbit anti-mouse CD31 and then treated with Alexa Fluor647-conjugated goat anti-rabbit antibody according to the manufacturer’s description (eBioscience). Nuclei were counterstained with 4′,6-diamidino-2-phenylindole (DAPI, Vector Laboratories Inc., Burlingame, CA). Images were taken using fluorescence microscopy. Three randomly selected microscopic fields were quantitatively analyzed on ImageJ software. Collagen in tumor tissues was detected by Masson Trichrome assay. The tumor slides were stained using a Masson Trichrome Kit (Saint Louis, MO, USA) according to the manufacturer’s instructions. Images were taken using light microscopy. Three randomly selected microscopic fields were quantitatively analyzed on ImageJ software.

### Flow Cytometric Evaluation of Immune Cell Populations

The immune cell population in the tumor tissues with various treatments was analyzed by flow cytometry. Cell suspensions from spleens or tumors were filtered, and red blood cells were lysed. For extracellular staining, the cells were incubated with the indicated combinations of antibodies (CD45, CD8, CD4, CD11b, Gr-1, F4/80, CD86, and CD206). For intracellular staining, the cells were fixed and permeabilized immediately after cell surface staining according to the manufacturer’s description (eBioscience), and combinations of antibodies (FoxP3) were added to cells in permeabilization buffer. All flow data were collected on a flow cytometer (FACS Calibur, BD Biosciences, USA). The data were analyzed using FlowJo software.

### Quantitative Real-Time PCR (RT-PCR) Assay

Total RNA was isolated from tumor using TRIzol reagent (Takara Bio, Shiga, Japan), and cDNA was reverse-transcribed by a one-step RT Kit (Takara Bio, Shiga, Japan). The resulting cDNA was used as a PCR template for determining the mRNA expression level using an SYBR-green quantitative PCR kit (Takara Bio, Shiga, Japan) with PCR. RT-PCR assays were performed for each sample in a final reaction volume of 20 µL, containing 10 µL SYBR-green fluorescent dye, 2 µL cDNA, and 50 pmol each of the forward and reverse primers (Servicebio, Wuhan, China). Primers for mouse interleukin (IL) 6, IL10 were purchased from Servicebio (Wuhan, China). Mouse GAPDH primers were used as an endogenous control. The RT-PCR was performed in triplicate for each group.

### Statistical Analysis

All values were expressed as the mean ± SD. Statistical analysis was performed using GraphPad Prism 6.0 (GraphPad Software, CA, USA). Differences between two groups were analyzed by Student’s t test. Differences for multi-groups were analyzed by one-way analysis of variance followed by Newman–Keuls post hoc test.

## Results and Discussion

### Ginsenoside Rh2 Engineered Lipid Bilayer and Liposomes with Better Stability

**Fig. 2:**
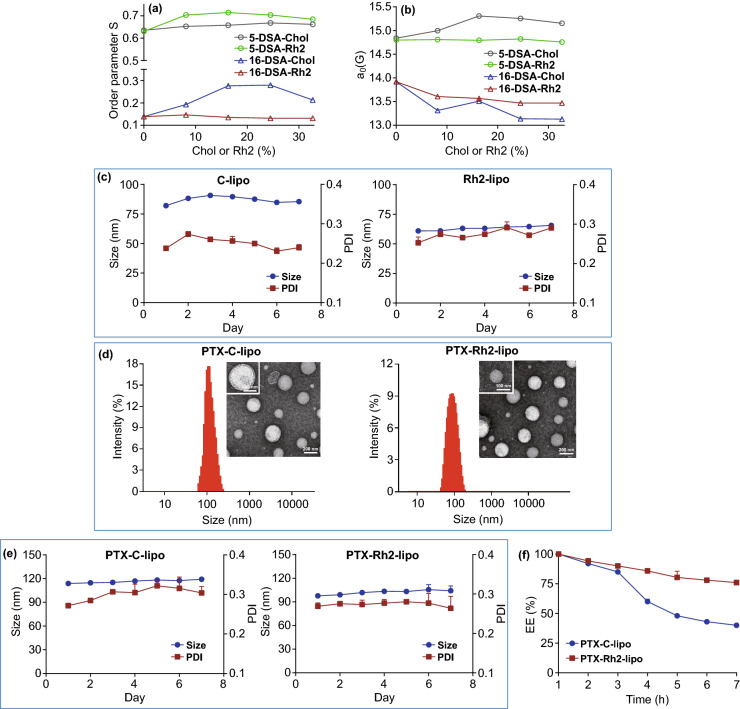
Rh2 worked as a membrane stabilizer, which keep the stability of liposomes. **a** Order parameter S of 5-DSA and 16-DSA measured as a function of cholesterol (Chol) or Rh2 concentration. **b** Isotropic hyperfine coupling constant *a*_0_ of 5-DSA and 16-DSA measured as a function of Chol or Rh2 concentration. **c** Stability of two blank liposomal formulations kept in 4 °C conditions monitored by dynamic light scattering (DLS) (*n* = 3; mean ± SD). **d** Size distribution of PTX-loaded C-lipo and Rh2-lipo was examined with DLS and TEM. **e** Stability of two PTX-loaded liposome formulations kept in 4 °C conditions monitored by DLS (*n* = 3; mean ± SD). **f** Drug leakage profiles of PTX loaded C-lipo and Rh2-lipo (*n* = 3; mean ± SD)

EPR spin label was used to investigate the modulating effect of Rh2 on the lipid bilayer of liposomes [38]. Higher-order parameter S indicates an ordered membrane arrangement with reduced fluidity and improved stability [39]. As shown in Fig. 2a, after cholesterol incorporation, the packing order near the polar head of the lipids (at 5-DSA position) did not change obviously, while a dramatic rise of S was observed in hydrophobic core (at 16-DSA position) compared with pure lipid, indicating that cholesterol mainly enhanced stability of lipid molecules in the hydrophobic region. The insertion of Rh2 could also inhibit the free movement of the lipid molecules, but Rh2 preferred to reduce the fluidity of polar head region rather than hydrophobic region of the lipids. Another parameter, isotropic hyperfine coupling constant *a*_0_ was measured to determine the hydrophobicity of the membrane [40]. A decrease of *a*_0_ indicates an increase in hydrophobicity at the spin label position. As shown in Fig. 2b, cholesterol and Rh2 insertion both presented a rising trend of hydrophobicity at bilayer center. However, cholesterol incorporation led to a great decrease in hydrophobicity in polar head group regions, while the change could be almost ignored after Rh2 incorporation. In most cases, lipid with higher hydrophobicity could decrease the penetration of oxygen and water, which otherwise would cause oxidative damage and induce the swelling of liposomes [41]. Therefore, it could be rationally concluded that Rh2 acts as a membrane stabilizer to enhance the endurance of liposomes against external environmental stresses. Further, stability test showed that both C-lipo and Rh2-lipo hold a stable particle size and PDI under storage condition (Fig. 2c).

Next, in order to figure out the encapsulation and stabilization capacity of Rh2 liposome, PTX was used as a model drug in this study. The mean particle sizes, PDI, and ζ-potentials of various formulations are listed in Table 1. Notably, the liposomes modified with Rh2 resulted in a much higher zeta potential than others. The loading of PTX did not reverse the trend. The loading of PTX into C-lipo and Rh2-lipo resulted in an increase in particle size, which were 118.01 and 99.03 nm, respectively (Table 1). This result was consistent with previous reports [42, 43]. The morphology of PTX-C-lipo and PTX-Rh2-lipo, observed by TEM, was homogeneously spheroids with moderate dispersity (Fig. 2d). EE and LE of PTX-Rh2-lipo were 91.3% and 5.6%, respectively, similar to those of PTX-C-lipo. Stability test was performed to qualify the capacity of preventing the drug from being damaged by external environment. Both PTX-C-lipo and PTX-Rh2-lipo hold a stable particle size and PDI under storage condition (Fig. 2e). Nevertheless, significant difference was observed between two liposomes in drug leakage test. As shown in Fig. 2f, compared with the rapid drug leakage in PTX-C-lipo, PTX-Rh2-lipo exhibited a steady behavior that cumulative leakage of PTX from the liposomes was less than 25% within 7 days, implying a good drug protection capacity. Besides, free Rh2 was essentially undetectable during the entire leakage study (data not shown).

**Table 1:** Characterization of different liposomes (*n* = 3; mean ± SD)

	Size (nm) ± SD	PDI ± SD	ZP (mV) ± SD	EE (%) ± SD	LE (%) ± SD
C-lipo	82.02 ± 1.54	0.23 ± 0.005	− 17.86 ± 0.32		
Rh2-lipo	60.95 ± 3.22	0.25 ± 0.014	− 37.13 ± 1.12		
PTX-C-lipo	118.01 ± 2.46	0.26 ± 0.011	− 21.86 ± 0.82	90.1 ± 1.6	6.4 ± 0.2
PTX-Rh2-lipo	99.03 ± 3.22	0.27 ± 0.014	− 39.21 ± 1.03	91.3 ± 2.1	5.6 ± 0.3

### Ginsenoside Rh2 Functionalized Liposomes with Longer Blood Circulation

**Fig. 3:**
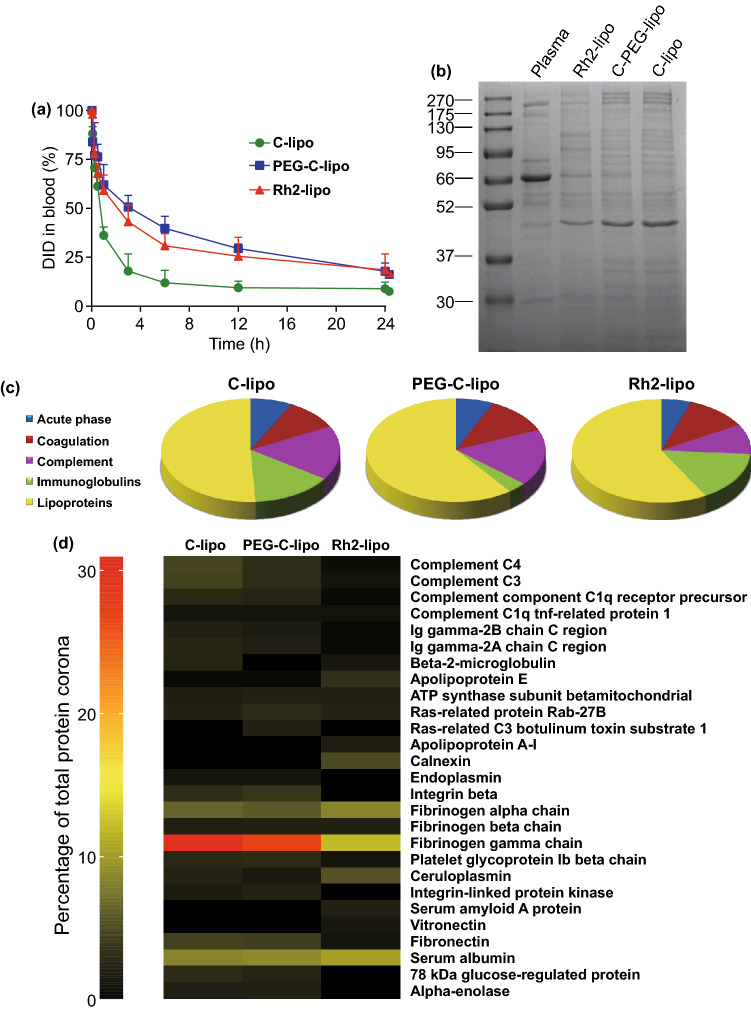
Rh2 worked as a long-circulating stealther, which specifically prolonged circulation behavior of liposomes. **a** Blood circulation profiles of C-lipo, PEG-C-lipo and Rh2-lipo (*n* = 3; mean ± SD). **b** Representative SDS-PAGE gel of protein coronas obtained from three liposomes following 1-h incubation with plasma. **c** Classification of protein corona components according to their function in biological processes and the protein number of each type (*n* = 2). **d** Heat map of the most abundant proteins in the protein corona. Only those proteins that constitute at least 1% of the protein corona on one of the liposomes are shown

To illustrate the long-circulating effect of Rh2-lipo, the blood circulation of Rh2-lipo was monitored following intravenous injection to healthy mice by determining the amount of residual fluorescence of DID for 24 h. As shown in Fig. 3a, similar to PEG-C-lipo, Rh2-lipo showed a specifically sustained circulation behavior. In addition, the *t*_1/2γ_ and area under plasma concentration–time curve (AUC) of DID were significantly increased in Rh2-lipo, which were about threefold higher than those of C-lipo (Table 2). As reported, the rapid clearance of liposomes is associated with an irreversible uptake by reticuloendothelial system (RES) [44]. Upon entering the biological media, liposomes were rapidly bounded with various proteins forming a PC, which would be recognized and captured by RES [45, 46]. Thus, in order to understand the stealth mechanism of Rh2-lipo, the composition of PC was analyzed and compared with other two liposomes. SDS-PAGE analysis showed that PC composition of Rh2-lipo was quite different from other two groups after incubating with plasma (Fig. 3b). It is noteworthy that PEG-C-lipo is coated with an almost identifiable protein pattern, compared with its un-PEGylated counterpart. To investigate the difference of the total protein composition, a semi-quantitative densitometry analysis of the protein bands was performed. The results indicated that Rh2-lipo, similar to PEG-C-lipo, was coated with significantly lower amount of PC than C-lipo (Fig. S1a, b). It is correspondent with the fact that PEG forms a steric barrier at the surface of nano-carriers to minimize their protein binding [47]. Specific type of the proteins in PC was further analyzed by nano-LC–MS/MS, where a total of 349 proteins were identified and grouped according to their functions. The results showed that compared with C-lipo, PEG-C-lipo and Rh2-lipo were coated with significantly fewer immunoglobulins and complement proteins and higher percent of lipoproteins belonging to dysopsonins (Fig. 3c). It has been verified that immunoglobulins and complement proteins are associated with opsonization and are the major target of RES. Therefore, the stealth effect of Rh2-lipo is possibly exerted by the reduction in opsonization and failure of RES recognition. Furthermore, the most abundant proteins composition in the PC showed the trend more clearly (Fig. 3d). Compared with C-lipo, PEGylation mainly changed the amount of proteins absorbed around liposomes, while Rh2 insertion changed the type of proteins thoroughly. More importantly, as shown in Fig. 3d, the amount of immunoglobulins and complement proteins, such as Ig gamma-2B chain C region, Ig gamma-2A chain C region, complement C3, complement C4, and complement component C1q receptor precursor, was decreased, while apolipoprotein E, which has been clearly reported to retard macrophages uptake in the blood, was increased in Rh2-lipo group [48]. Interestingly, fibrinogen gamma chain (48 kDa), a coagulation which is the major protein in the corona of C-lipo and can be assigned to the band between 37 and 52 kDa in the SDS–PAGE, was strongly depleted on the surface of Rh2-lipo. Although fibrinogen gamma chain was also found to be enriched in the PC of nano-carriers in previous publications [49], there is no evidence that it could impact the in vivo circulation of a nano-carrier.

**Table 2:** PK parameters after liposomes administration (n = 3; mean ± SD)

	C-lipo	PEG-C-lipo	Rh2-lipo
*t*_1/2γ_ (h)	7.34 ± 2.41	23.49 ± 5.14**	21.98 ± 6.77*
AUC_0-∞_ (μg/mL × h)	471.46 ± 94.77	1418.42 ± 172.33**	1394.60 ± 432.1**

*P < 0.05, **P < 0.01

Hence, Rh2-lipo is similar to PEGylated liposomes in prolonging the liposome circulation in vivo. However, the long-acting mechanism they functioned through was different, which could be summarized as follows: Firstly, PEGylation mainly changed the amount of proteins absorbed around liposomes, while Rh2-lipo changed the type of proteins thoroughly. Secondly, although both PEG-C-lipo and Rh2-lipo were coated with fewer proteins belong to opsonization than C-lipo, PEG-C-lipo mainly reduced the affinity of immunoglobulins, while Rh2-lipo decreased the adsorption by complement proteins (Fig. 3c). Thirdly, apolipoprotein E was increased in Rh2-lipo group but not observed in PEG-C-lipo (Fig. 3d), which would help to retard macrophages uptake toward liposomes in the blood.

### Ginsenoside Rh2 Functionalized Liposomes with Active Targeting Effect by Interacting with GLUT

**Fig. 4:**
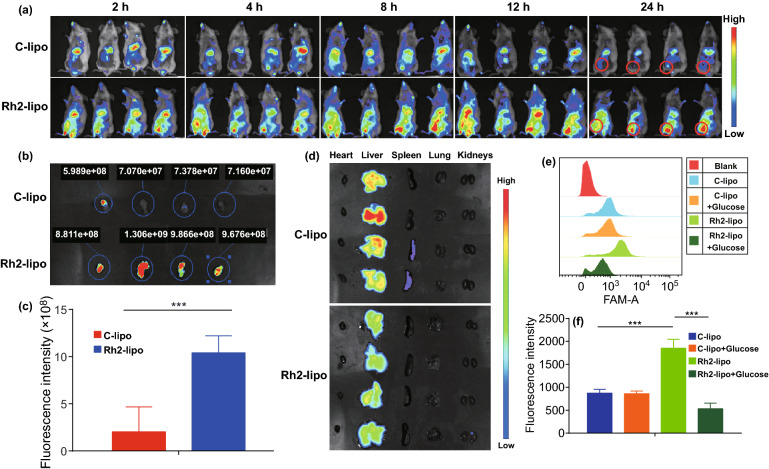
Rh2 worked as an active targeting ligand, which enhanced liposomes to accumulate in tumor by the interaction with GLUT. **a** Biodistribution of the DID-labeled C-lipo and Rh2-lipo after intravenous injection in 4T1 tumor-bearing mice. **b** Fluorescence images of excised tumors at 24 h. **c** Fluorescence intensity in tumors from different groups (*n* = 4; mean ± SD). ***P < 0.001. **d** Fluorescent images of dissected organs of mice sacrificed 24 h. **e** Cellular uptake of FAM-labeled Rh2-lipo and its competition with glucose using flow cytometry analysis. **f** Ratio of the relative fluorescence intensity of cellular uptake from different groups (*n* = 3; mean ± SD). ***P < 0.001

The targeting effect of Rh2-lipo was evaluated in a 4T1-orthotopic tumor model (Fig. 4a). After DID-labeled liposomes were given through the tail vein, the Rh2-lipo group exhibited a much stronger fluorescence signal in the tumor region at all-time points compared with conventional liposome group, indicating that Rh2-lipo could significantly facilitate liposomes to accumulate effectively at the tumor site. The tumor targeting ability of Rh2-lipo was further verified by ex vivo imaging of the tumors after 24 h, as shown in Fig. 4b, c. Moreover, the spleen and liver of Rh2-lipo group showed a much weaker fluorescence signal, which was owing to the slow clearance feature of Rh2-lipo (Fig. 4d). To further explain the mechanism of prominent targeting effect, competition assay was performed and the cellular uptake of FAM-loaded Rh2-lipo on 4T1 cells was analyzed by flow cytometry in vitro (Fig. 4e, f). In correspondence with previous results, Rh2-lipo exhibited higher fluorescence intensity than C-lipo in the cellular uptake assay. After adding excess free glucose to saturate GLUT, the uptake of Rh2-lipo was obviously decreased, while no impact was observed for C-lipo, suggesting that the uptake of Rh2-lipo could be evidently facilitated via GLUT which was overexpressed on tumor cells. Also, observation by confocal microscope showed a same result (Fig. S2). Therefore, the interaction of GLUT with Rh2 promoted the retention of Rh2-lipo and facilitated its entry into tumor cells.

### In Vitro/In Vivo Anti-tumor Activity of PTX Loaded Rh2 Liposomes

**Fig. 5:**
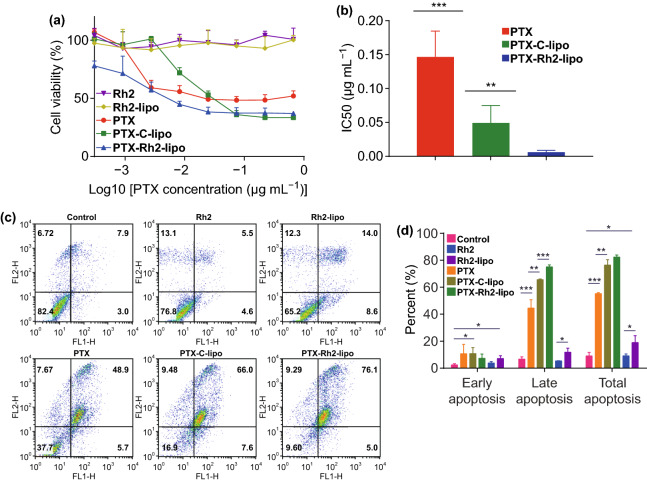
**a** Cytotoxicity of different groups against 4T1 cells (*n* = 4, mean ± SD). **b** IC 50 values of different PTX formulations. **P < 0.01 and ***P < 0.001. **c** Representative scatter plots of Annexin V/PI analysis of 4T1 cells after drug treatments. **d** Percentage of cells with early, late, and complete apoptosis (*n* = 3; mean ± SD). *P < 0.05, **P < 0.01 and ***P < 0.001

To examine the cytotoxicity of PTX-loading Rh2-lipo against tumor cells, MTT and apoptosis assay were performed on 4T1 cells in vitro. As shown in Fig. 5a, no significant inhibition of Rh2 and Rh2-lipo was found on the tumor cell growth at test concentrations. Free PTX inhibited the tumor cell growth in a concentration-dependent manner, while the effect could be enhanced by being loaded into C-lipo. In contrast, PTX-Rh2-lipo showed the strongest inhibition ability, whose IC50 was only 1/8 of that of PTX-C-lipo (Fig. 5b). Therefore, it could be concluded that Rh2 incorporation significantly promoted the anti-tumor efficacy of conventional PTX liposomes. Apoptosis assay was performed with Annexin V/PI double staining. As shown in Fig. 5c, d, PTX-Rh2-lipo led to more than 80% apoptosis of 4T1 cells. Free PTX showed less than 60% cell apoptosis, while PTX-C-lipo increased the total apoptosis to about 80%, which was similar to PTX-Rh2-lipo group. However, due to the synergy effect between Rh2 and PTX, the percent of late apoptosis induced by PTX-Rh2-lipo was greatly stronger than that of PTX-C-lipo. It was also noted that blank Rh2-lipo alone also had apoptosis efficacy toward tumor cells, whereas Rh2 itself had little apoptotic capacity, indicating that the preparation of Rh2-lipo was also critical to the anti-tumor effects of PTX-Rh2-lipo. Thereby, Rh2 acted as more than a membrane material in this formulation, which was also an adjuvant drug.

**Fig. 6:**
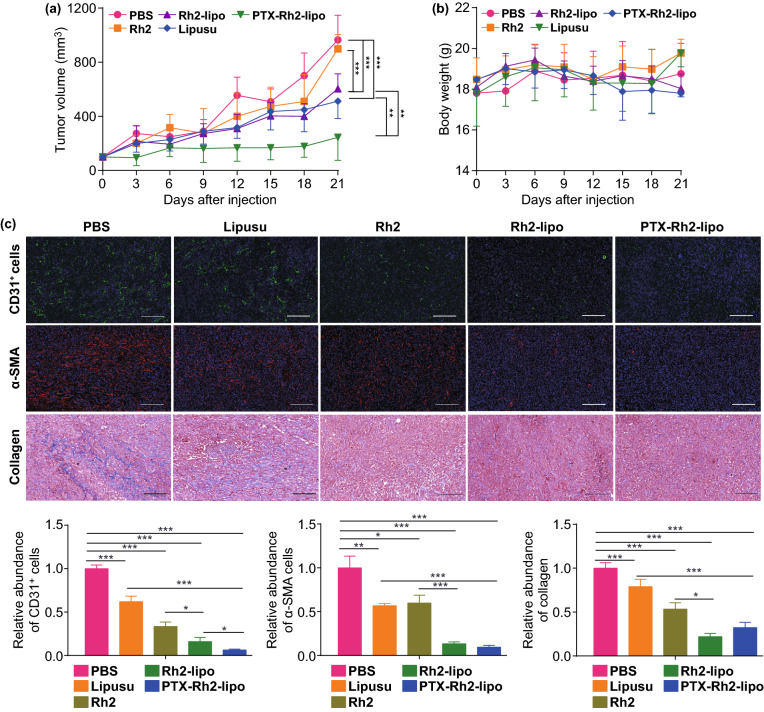
Rh2 worked as a chemotherapy adjuvant, which suppressed tumor growth and remodeled structure of TME. **a** 4T1-orthotopic tumor growth curve during PTX loaded Rh2-lipo treatments (*n* = 6; mean ± SD). *P < 0.05, **P < 0.01 and ***P < 0.001. **b** Body weight of 4T1-orthotopic tumor model mice treated by different formulations (*n* = 6; mean ± SD). **c** Blood vessels in 4T1 tumors, marked with CD31 antibody (green), were normalized in structure and reduced in expression after treatment; TAFs in tumors, marked with α-SMA antibody (red), were significantly decreased after treatment; Collagen fibers, marked with Masson’s Trichrome, were significantly decreased after treatment. Scale bar = 100 μm. *P < 0.05, **P < 0.01 and ***P < 0.001, (*n* = 3; mean ± SD)

Lipusu, also known as *Paclitaxel Liposome for Injection*, manufactured by Luye Pharma Group and Nanjing Sike Pharmaceutical, has been clinically approved in China as second-line chemotherapy for breast cancer. Therefore, it was selected as a control group for comparing the anti-tumor efficiency with PTX-Rh2-lipo in vivo. The anti-tumor effect of PTX-Rh2-lipo was studied on a 4T1-orthotopic tumor model with PTX dosage of 10 mg kg^−1^. After orthotopic implantation, the mammary tumor treated with PBS grew progressively and increased approximately tenfold in volume from day 0 to day 21. As shown in Fig. 6a, no obvious difference was observed between free Rh2 treatment and PBS group. However, when Rh2 was inlaid in lipid to form liposomes, it magically reduced tumor growth to the extent that was comparable to that of Lipusu. This phenomenon was inconsistent with the results in vitro, indicating that Rh2-lipo could inhibit tumor development through other pathways rather than a direct cytotoxicity to tumor cells. Of note, PTX-Rh2-lipo led to great tumor growth retardation, displaying a remarkable inhibition of tumor size at the end point (PTX-Rh2-lipo vs Lipusu). The wonderful therapeutic effect of this formulation was further well supported by the TUNEL apoptosis assay in Fig. S3. All of the treatments were well tolerated with no abnormal physical signs detected in all treated mice. As shown in Figs. 6b and S4, the body weights and organ weights showed no significant change in all the groups of treatment. Though histopathological diagnosis of major organs showed that some organs were invaded by inflammatory cells and metastatic cancer cells, causing different degrees of tissue necrosis (Fig. S5), PTX-Rh2-lipo could obviously alleviate this tissue damage and necrosis.

### Ginsenoside Rh2 Functionalized Liposomes with TME Remodeling Effect

A numerous evidence has shown that the hypoxic tumor environment not only results in a microenvironment that suppresses the immune response and supports tumor cell survival, but also acts as moats and walls for preventing the attack of anti-cancer therapeutics into the core of tumor [50]. Therefore, we further investigated the impact of this special formulation on remodeling the TME by studying the distribution of vessel, expression of TAF and collagen as well as the immune response. As shown in Fig. 6c, the vessels (green fluorescence) treated by PBS were abundant and distributed randomly with a thin and elongated morphology that can greatly restrain the infiltration of drugs from vessels. Under the high pressure in the TME, tumor vessels treated by Lipusu were still crushed. Single therapy by free Rh2 or Rh2-lipo showed a much greater anti-angiogenic activity than Lipusu, in which the remaining vessels displayed an open and round morphology. Weakest fluorescence was observed after PTX-Rh2-lipo treatment, demonstrating that the formulation induced the strongest blood vessel network normalization effect in the tumor. The TAF, marked by α-SMA staining, showed that Lipusu and Rh2-lipo alone could partially attenuate the fibroblast population in the tumor tissue, while PTX-Rh2-lipo almost completely depleted the amount of α-SMA-positive cells, indicating that the loading of PTX into Rh2-lipo could also eliminate TAFs effectively. Stroma, described by the content and morphology of collagen (Masson’s Trichrome staining) [51], exhibited thick and overspread fibrous structures after being treated with PBS. Though other groups all down-regulated the expression of collagen, PTX-Rh2-lipo showed the strongest collagen inhibition effect. All in all, the results demonstrated that PTX-Rh2-lipo elicited a significant improvement of the structure of TME.

**Fig. 7:**
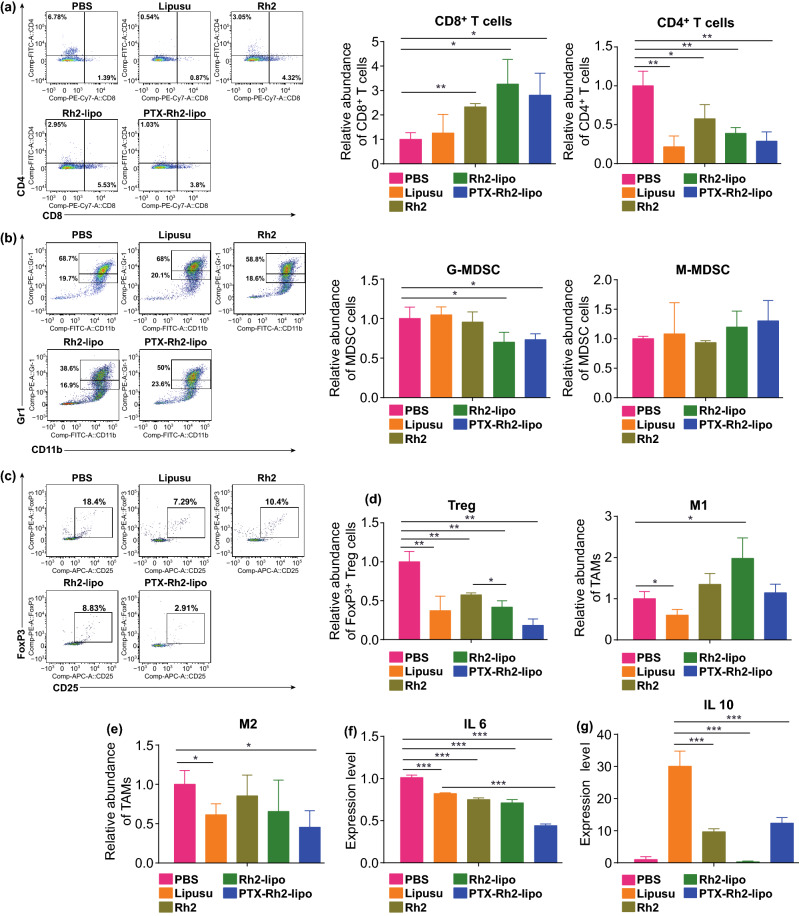
Rh2 worked as a chemotherapy adjuvant, which reversed immunosuppressive TME. **a** Relative abundance of CD4^+^, CD8^+^ cells in tumor tissues was detected by flow cytometry. **b** Flow cytometry gating and histograms analysis of CD11b^+^/Gr-1^+^ MDSC cells in tumors. Double positive cells contain two populations, including Gr-1^high^CD11b^+^ granulocytic (G-MDSC) and Gr-1^int^CD11b^+^ monocytic (M-MDSC) MDSC subsets. **c** Flow cytometry gating and histogram analysis of FoxP3^+^ T regulatory cells in tumors. **d**, **e** The percentages of TAM populations with specific macrophage markers M1-type (CD11b +/F4/80 +/CD86 +) and M2-type (CD11b +/F4/80 +/CD206 +) in tumor tissues were detected by flow cytometry. **f**, **g** Tumor cytokine IL6 and IL10 level were detected by using RT-PCR. *P < 0.05, **P < 0.01 and ***P < 0.001, (*n* = 3; mean ± SD)

The reversion of suppressive immune state in TME was also analyzed using flow cytometry one day following seven times of treatments. Figure 7a shows that the infiltration of CD8^+^ T cells (CD45^+^/CD8^+^) in the tumors treated with PTX-Rh2-lipo was higher than that of PBS and Lipusu groups. Rh2 or Rh2-lipo could also effectively increase the number of CD8^+^ T cells, suggesting the immunological enhancement role of Rh2. However, the CD4^+^ T cells (CD45^+^/CD4^+^) in all treatment groups were less than PBS group (Fig. 7a). T regulatory cells (CD45^+^/CD4^+^/CD25^+^/FoxP3^+^), one of the CD4^+^ T cells that inhibit the immune response in the tumor, were significantly suppressed in all treatment groups (Fig. 7c). Another kind of immune cells with immune suppression function, myeloid-derived suppressor cells (MDSC, CD11b^+^/Gr-1^+^), was also detected. Figure 7b shows that the number of granulocytic myeloid-derived suppressor cells (G-MDSC) was significantly decreased in the tumors treated with Rh2-lipo and PTX-Rh2-lipo, while G-MDSC held the same level as control group in Rh2 or Lipusu group. It indicated that Rh2 could not display G-MDSC suppression effect unless be loaded and delivered into the tumor site by Rh2-lipo. Figures 7d, e and S6 indicate that the numbers of M2 tumor-associated macrophages (TAM) (CD45^+^/F4/80^+^/CD206^+^) were significantly reduced in PTX-Rh2-lipo group, while the numbers of M1 TAM (CD45^+^/F4/80^+^/CD86^+^) had no change. Overall, PTX-Rh2-lipo treatment was more immune-active than Lipusu in reducing suppressor cells and enhancing CD8^+^ T cells infiltration in TME. To further understand the mechanisms underlying the reversion of immune suppression mediated by PTX-Rh2-lipo, the expression of two immunosuppressive cytokines IL 6 and IL10 that were typically derived from tumor was evaluated [52]. As shown in Fig. 7f, all treatment groups down-regulated the level of IL6. Among which, PTX-Rh2-lipo had the strongest effect. Lipusu treated tumors exhibited an explosive growth in IL10 which was effectively prevented by the treatment of PTX-Rh2-lipo, suggesting that the Rh2-lipo system could reverse the immunosuppressive environment in TME.

## Conclusions

In summary, the main objective of this study was to develop a novel and unique Rh2 liposomal system to possibly address the limitations of current liposome formulations, such as (1) the difficulty to improve the progress of TME; (2) the disadvantages of the PEG utilization to extend circulation time of liposomes; (3) the complicated fabrication process and low targeting efficiency of ligand-modified liposomes; (4) the problems for cholesterol as an ingredient of traditional liposomes. In this novel Rh2-lipo-based nano-carrier, ginsenoside Rh2 could serve as a multifunctional membrane material to stabilize the structure and prolong the blood circulation of liposomes. It also worked as an active ingredient to synergically enhance the efficacy of anti-cancer drugs by remodeling tumor-associated microenvironment and stimulating the immune system. Although only PTX was selected as the model drug in this study, the application could be extended to other anti-cancer agents including doxorubicin, docetaxel, cisplatin, and irinotecan (data not shown). This Rh2-lipo system innovatively challenged the irreplaceable position of cholesterol as liposome component as well, which would provide another innovative potential system with multiple functions for anti-cancer drug delivery.

## Electronic Supplementary Material

Below is the link to the electronic supplementary material.

Supplementary material 1 (PDF 736 kb)
