# Supplementary material for: One Stone Four Birds: A Novel Liposomal Delivery System Multi-functionalized with Ginsenoside Rh2 for Tumor Targeting Therapy
Source: Nanomicro Lett. 2020 Jun 16;12:129. doi: 10.1007/s40820-020-00472-8 (PMC7770862; doi:10.1007/s40820-020-00472-8)
Supplement: Supplementary file 1 — Supplementary material 1 (PDF 736 kb) [file 40820_2020_472_MOESM1_ESM.pdf]

Supporting Information for

One Stone Four Birds— A Novel Liposomal Delivery System Multi-Functionalized with Ginsenoside Rh2 for Tumor Targeting Therapy

Chao Hong[1], Jianming Liang[1, 2], Jiaxuan Xia[1], Ying Zhu[3], Yizhen Guo[1], Anni Wang[1], Chunyi Lu[4], Hongwei Ren[1], Chen Chen[1], Shiyi Li[1], Dan Wang[1,5], Huaxing Zhan[5], Jianxin Wang[1, 6, *]

[1]Department of Pharmaceutics, School of Pharmacy, Fudan University & Key Laboratory of Smart Drug Delivery, Ministry of Education, Shanghai 201203, People's Republic of China

[2]Institute of Tropical Medicine, Guangzhou University of Chinese Medicine, Guangzhou 510006, People's Republic of China

[3]Institute of Clinical Pharmacology, Guangzhou University of Traditional Chinese Medicine, Guangzhou 510006, People's Republic of China

[4]School of Pharmacy, Shanghai Jiao Tong University, Shanghai 200240, People's Republic of China

[5]Shanghai Ginposome Pharmatech Co., Ltd, Shanghai 201600, People's Republic of China

[6]Institute of Integrated Chinese and Western Medicine, Fudan University, Shanghai 200040, People's Republic of China

*Corresponding author. E-mail: jxwang@fudan.edu.cn (Jianxin Wang)

Supplementary Figures

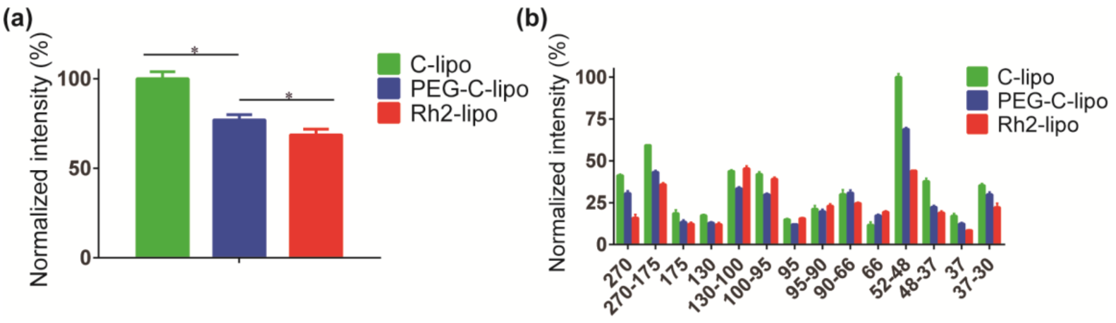

Fig. S1 a Histograms representing the total lane intensity of proteins in SDS-PAGE gel recovered from different groups (n = 2; mean ± SD). *P < 0.05. **b** Lane intensity results of fourteen molecular weight ranges in SDS-PAGE gel

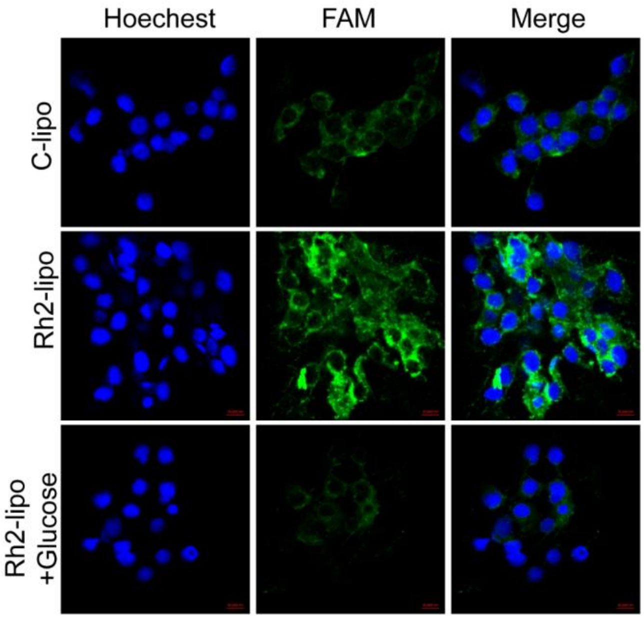

Fig. S2 Qualitative cellular uptake of FAM-labeled liposomes in 4T1 cells. The cells were incubated with 500 ng mL^{-1} FAM-loaded liposomes at 37 °C for 4 h (n = 3; mean ± SD); scale bar = 20 µm

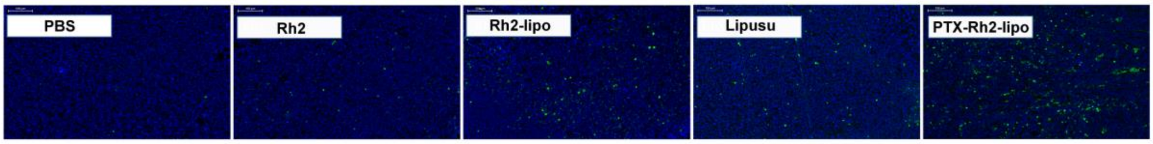

Fig. S3 TUNEL-positive cells in tumor sections excised from the mouse after drug treatments

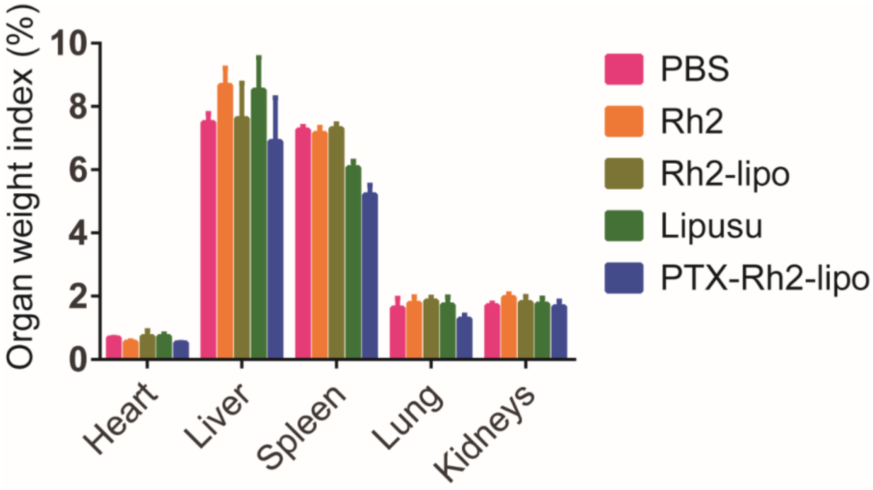

Fig. S4 Organ weight indexes at the termination of anti-tumor experiments

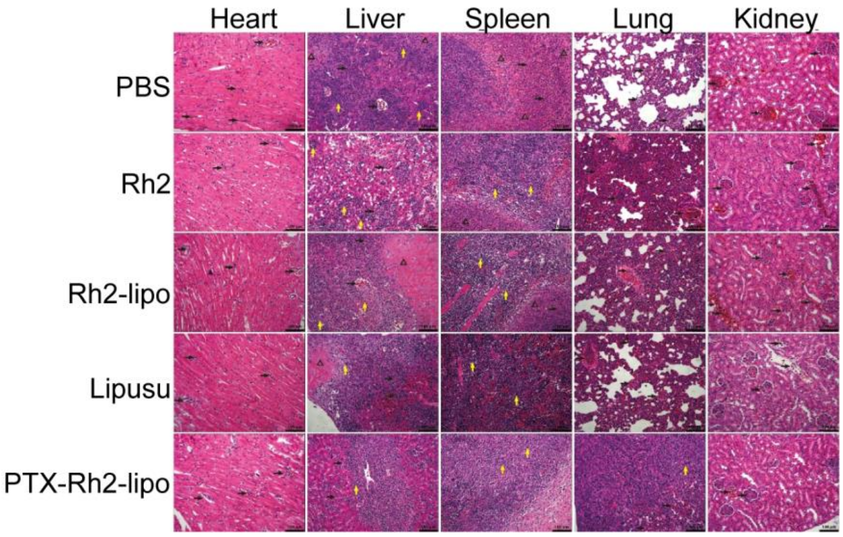

Fig. S5 Representative H&E stained sections of organs. ➡ Inflammatory cells; ⬆ Metastatic cancer cells; △ Necrotic cells

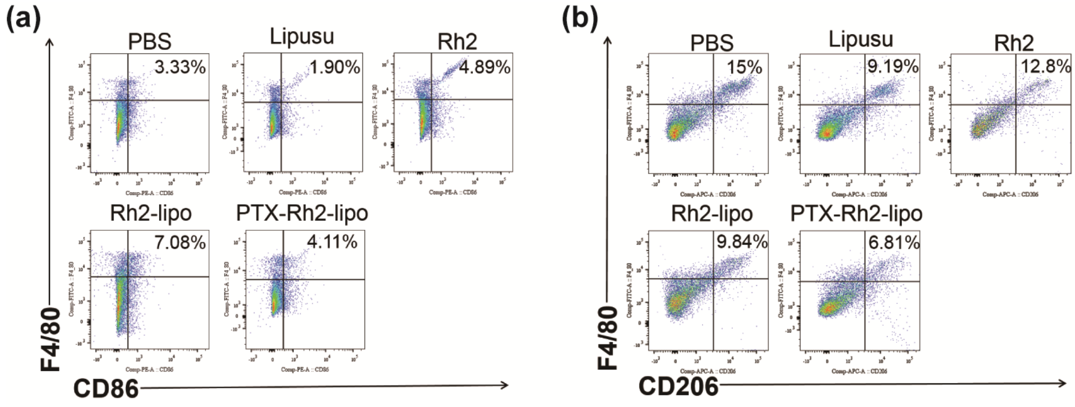

Fig. S6 The percentages of TAM populations with specific macrophage markers M1-type ($CD11b^+/F4/80^+/CD86^+$) (**a**) and M2-type ($CD11b^+/F4/80^+/ CD206^+$) (**b**) in tumor tissues were detected by flow cytometry
